# CDK9 and H2B Monoubiquitination: A Well-Choreographed Dance

**DOI:** 10.1371/journal.pgen.1002860

**Published:** 2012-08-02

**Authors:** Steven A. Johnsen

**Affiliations:** 1 Department of Tumor Biology, University Medical Center Hamburg-Eppendorf, Hamburg, Germany; 2 Department of Molecular Oncology, University of Göttingen Medical Center, Göttingen, Germany; Albert Einstein College of Medicine, United States of America

Transcriptional regulation in eukaryotes requires the transcriptional machinery to negotiate the complexities of DNA packaged into chromatin. Specific modifications of the core histone proteins serve to regulate transcription and ensure that genes are expressed at the right place and the right time [1]. Not surprisingly, the interaction between the transcriptional apparatus and chromatin is a tango in which the partners are in a close embrace. Cyclin-dependent kinase 9 (CDK9) and its orthologs control both transcription and transcription-coupled chromatin modifications in a variety of species [2], [3]. However, the mechanisms that intertwine CDK9 function with chromatin appear to be distinct in different organisms. A new manuscript in this issue of *PLoS Genetics* sheds light onto how Cdk9 function is interconnected with the monoubiquitination of histone H2B in the fission yeast *Schizosaccharomyces pombe* [4].

## The Dance Partners: CDK9 and H2Bub1

CDK9 controls transcriptional elongation, transcription-coupled mRNA processing, and histone modification [2], [5]. It accomplishes this by phosphorylating a number of central transcriptional regulatory proteins, including RNA Polymerase II (RNAPII) (reviewed in [6]), Suppressor of Ty-5 (Spt5; or its metazoan ortholog SUPT5H) [7], [8], Rad6 (metazoan UBE2A) [9], [10], and Negative Elongation Factor-E (NELF-E) [11], [12]. Notably, phosphorylation of Ser2 within the repeated YSPTSPS heptapeptide motif of the RNAPII carboxy-terminal domain (CTD) and Thr1 within the Spt5 C-terminal repeat (CTR) are conserved across eukaryotes and responsible for various aspects of CDK9 function.

One post-translational histone modification emerging as a key player in numerous processes is histone H2B monoubiquitination (H2Bub1). In mammals this modification appears to serve as an important tumor suppressor [13]. H2Bub1 is associated with the transcribed region of active genes [14], [15] and promotes transcriptional elongation in vitro [16]. Interestingly, the trimethylation of histone H3 lysine 4 (H3K4me3) and lysine 79 (H3K79me3) also depend on H2Bub1 17–19, although its importance to mammalian H3K4me3 may be more limited [20], [21].

## New Insights into the CDK9–H2Bub1 Tango

The new study by Sansó et al. has unraveled additional details about the complex dance between the fission yeast ortholog of CDK9 (spCdk9, also called Pch1) and H2Bub1 [4]. The authors utilized a genome-wide approach to characterize the effects of a loss of H2Bub1 on RNAPII occupancy and mRNA levels and observed a surprising disconnect: while RNAPII occupancy was significantly impacted at the majority of active genes, only a subset were affected at the mRNA level. In the absence of H2Bub1, RNAPII levels within the transcribed region decreased, and its typical accumulation at the 3′ end of yeast genes was even more pronounced. Consistent with a potential role in transcriptional elongation, Sansó et al. show that H2B monoubiquitination depends on spCdk9 activity, but not on the related RNAPII CTD kinase Lsk1. They further establish that, unlike in human cells [22], [23], this effect is independent of RNAPII CTD phosphorylation and instead requires spCdk9-mediated Spt5 phosphorylation. However, the relationship between CDK9 and H2Bub1 is not a linear pathway. In fact, blocking H2B monoubiquitination by replacing the ubiquitinated lysine in H2B with an arginine (K119R) leads to decreased recruitment of spCdk9 and reduced levels of Spt5 phosphorylation.

Does this mean that spCdk9 and H2Bub1 play opposing roles in fission yeast? Phenotypic studies suggest that this may be at least partially true. For example, while H2B K119R mutation or deletion of the *S. pombe* H2B ubiquitin ligase Brl2 both lead to septation defects, these can be rescued by blocking spCdk9. Furthermore, compound mutants of Spt5 and Set1 suggest that the phenotypic effects of H2Bub1 loss may be due to multifaceted downstream effects of H2Bub1 on both Spt5 (through spCdk9 recruitment) and H3K4me3 (through Set1). Further support for a homeostatic feedback mechanism is provided by studies of RNAPII occupancy in fission yeast with reduced spCdk9 activity. Consistent with opposing roles of H2Bub1 and spCdk9, the authors observed increased RNAPII occupancy in transcribed regions and decreased occupancy at gene 3′ ends. Importantly, the combined mutation of spCdk9 and H2Bub1 rescued the H2B K119R mutation and displayed RNAPII occupancy similar to mutant spCdk9 alone.

**Figure 1:**
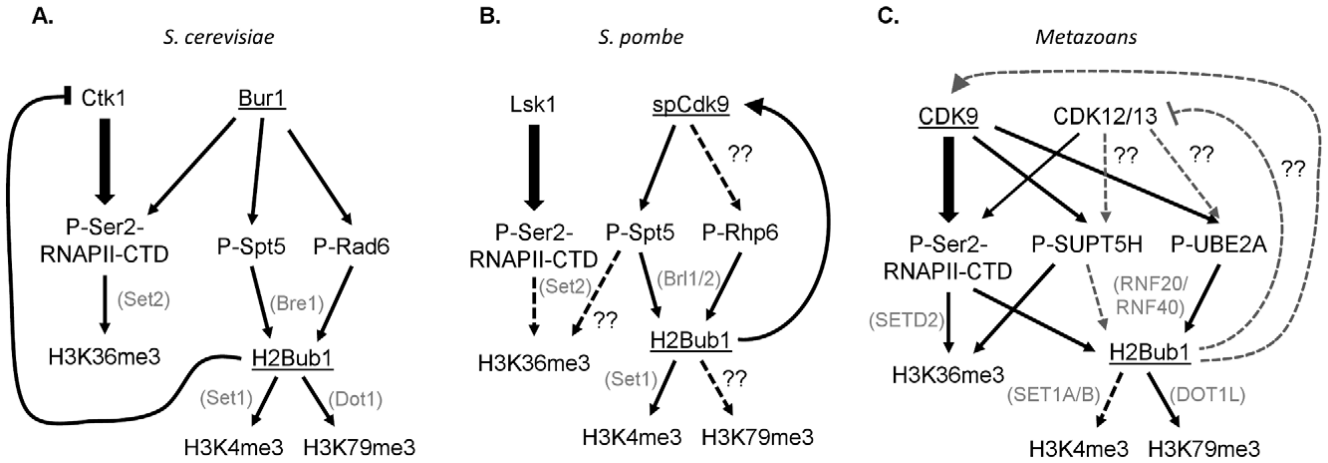
Interconnections between CDK9 orthologs, their substrates, and downstream histone modifications in yeast and humans. The CDK9 orthologs Bur1 in budding yeast (*S. cerevisiae*) and spCdk9 in fission yeast (*S. pombe*) are most closely related to human CDK9, while Ctk1 and Lsk1 are most homologous to CDK12 and 13. (A, B) In yeast the primary Ser2 RNAPII CTD kinases are Ctk1 and Lsk1. Furthermore, Spt5 phosphorylation, rather than Ser2 phosphorylation, is essential for H2Bub1 and its downstream histone modifications H3K4me3 and H3K79me. (C) In metazoans the separation of Ser2 and SUPT5H phosphorylation is less clear. Moreover, Ser2 phosphorylation is required for H2Bub1.

These results demonstrate the intricacy and complexity of the choreographed tango between CDK9 orthologs and chromatin, and illustrate significant differences between yeast and metazoans. This study shows that although H2Bub1 requires Cdk9 activity in fission yeast, Bur1 in budding yeast [24], and CDK9 in metazoans [5], [9], [20], [22], a different specific mechanism may operate in each species (Figure 1). In both yeasts, Spt5 appears to be the major Bur1/spCdk9 substrate responsible for controlling H2B monoubiquitination, and this occurs in a RNAPII CTD–independent manner [4], [8]. In human cells the situation appears more complex and involves the parallel effects of CDK9-dependent phosphorylation of the RNAPII CTD [20], [22], [23], as well as UBE2A [9] and probably also SUPT5H in some systems [5], [25]. The Sansó et al. study thus underscores the complexity of CDK9 function and the tight bilateral communication between chromatin and transcription. Further analyses will be necessary to address the various differences in CDK9 function in yeast and metazoans. For example, the existence of NELF-E and the prevalence of promoter proximal RNAPII pausing in metazoans likely represent key transcriptional elongation regulatory mechanisms that yeast lack. Furthermore, two additional closely related CDK9 orthologs, CDK12/CRKRS and CDK13/CDC2L5, have been identified and implicated in metazoan transcriptional elongation [26], [27]. Thus, determining the functions of the various CDK9 orthologs and identifying their substrates, and the interaction of these substrates with H2Bub1 and other chromatin modifications, represents an important challenge.
